# YOLOv4-Based CNN Model versus Nested Contours Algorithm in the Suspicious Lesion Detection on the Mammography Image: A Direct Comparison in the Real Clinical Settings

**DOI:** 10.3390/jimaging8040088

**Published:** 2022-03-24

**Authors:** Alexey Kolchev, Dmitry Pasynkov, Ivan Egoshin, Ivan Kliouchkin, Olga Pasynkova, Dmitrii Tumakov

**Affiliations:** 1 Department of Applied Mathematics and Informatics, Mari State University, Ministry of Education and Science of Russian Federation, 1 Lenin Square, Yoshkar-Ola 424000, Russia; kolchevaa@mail.ru (A.K.); passynkov@mail.ru (D.P.); olgaved@inbox.ru (O.P.); 2 Department of Radiology and Oncology, Mari State University, Ministry of Education and Science of Russian Federation, 1 Lenin Square, Yoshkar-Ola 424000, Russia; 3 Department of Fundamental Medicine, Mari State University, Ministry of Education and Science of Russian Federation, 1 Lenin Square, Yoshkar-Ola 424000, Russia; 4 Institute of Computational Mathematics and Information Technologies, Kazan Federal University, 18 Kremlevskaya St., Kazan 420008, Russia; dtumakov@kpfu.ru; 5 Department of Diagnostic Ultrasound, Kazan State Medical Academy—Branch Campus of the Federal State Budgetary Educational Institution of Further Professional Education “Russian Medical Academy of Continuous Professional Education”, Ministry of Healthcare of the Russian Federation, 36 Butlerov St., Kazan 420012, Russia; 6 Department of General Surgery, Kazan Medical University, Ministry of Health of Russian Federation, 49 Butlerov St., Kazan 420012, Russia; hirurgivan@mail.ru

**Keywords:** mammography, breast cancer, nested contours algorithm, convolutional neural network, YOLOv4

## Abstract

Background: We directly compared the mammography image processing results obtained with the help of the YOLOv4 convolutional neural network (CNN) model versus those obtained with the help of the NCA-based nested contours algorithm model. Method: We used 1080 images to train the YOLOv4, plus 100 images with proven breast cancer (BC) and 100 images with proven absence of BC to test both models. Results: the rates of true-positive, false-positive and false-negative outcomes were 60, 10 and 40, respectively, for YOLOv4, and 93, 63 and 7, respectively, for NCA. The sensitivities for the YOLOv4 and the NCA were comparable to each other for star-like lesions, masses with unclear borders, round- or oval-shaped masses with clear borders and partly visualized masses. On the contrary, the NCA was superior to the YOLOv4 in the case of asymmetric density and of changes invisible on the dense parenchyma background. Radiologists changed their earlier decisions in six cases per 100 for NCA. YOLOv4 outputs did not influence the radiologists’ decisions. Conclusions: in our set, NCA clinically significantly surpasses YOLOv4.

## 1. Introduction

Breast cancer (BC) still remains the one of the most important problems in global oncology, and in 2018 it shared the first incidence rate place with lung cancer (11.6% of all malignancies), despite the lower mortality (6.6% of all cancer-related deaths). At the same time, BC is the most frequently diagnosed malignancy in women (24.2% of all malignancies) associated with the largest cancer-related mortality (15.0%) [1]. The latter requires the development of strategies to decrease BC-related mortality, among which is early BC detection via the help of the population-wide mammographic screening programs. It was shown that a regular (every 1–2 years) invitation for mammography screening in women 50–69 years old (of which only 60% invited actually underwent the mammography) was associated with a 25% reduction of BC-related mortality risk (relative risk (RR): 0.75; 95% with confidence interval (CI): 0.69–0.81). In women of this age group who actually performed the mammography, the reduction was 38% (RR: 0.62; 95% CI: 0.56–0.69) [2]. However, in women aged 40–44 and 45–49, BC-related mortality risk reduction was less pronounced, and the Working Group on the Assessment of BC Screening of the International Agency for Research on Cancer (IARC) decided that the strength of the evidence that such screening reduces BC-related mortality is only limited [3].

The latter phenomenon is apparently due to the high prevalence of the dense breast parenchyma (C-D types according to the American College of Radiology (ACR) 2013) that lowers the sensitivity of the mammography to 50.0–68.1% (compared to 85.7–88.8% for the fatty breast parenchyma ACR A) [4,5]. At the same time, it was shown that the dense parenchyma may be associated with the increased RR in BC diagnosis (4.6, 95% CI: 1.7–12.6—in premenopausal women and 3.9, 95% CI: 2.6–5.8—in postmenopausal women). This is not surprising, because the dense breast is usually associated with the fibrocystic disease, of which the proliferative forms are the obligatory pre-cancer or associated with the other precancerous conditions [6].

One more problem in practical mammography is the difficulty to identify visually the small and atypical BC appearances, and these BC types are usually missed and diagnosed later in a more advanced stage that worsens the prognosis.

To overcome these problems, different approaches are being developed. One of the approaches corresponds to the mammography image processing by the computer-aided detection (CAD) systems of various designs.

At present, the machine learning and deep learning-based methods for image processing are the most interesting methods for CAD design [7,8]. Machine learning methods with supervised learning allow creating models that recognize various lesion types in mammography images. Deep learning (DL) networks have the potential to be used in the automated screening, staging of diseases, predicting the treatment effect, and disease outcome [9,10]. CAD systems based on machine learning methods such as support vector machine, naive Bayes, random forest, and logistic regression [11,12,13] usually depend on the handcrafted feature extraction step and are commonly used for breast cancer detection and classification. These methods show good accuracy (80–95%), but the false-positive error rate in the proposed systems is very high, and the scientific community is paying more attention to approaches that will reduce this rate [14].

To obtain the best results, it is often necessary to take into account the specific radiomic features of the analyzed image. This is especially important for the X-ray images that have a summation character significantly complicating both detection and discrimination of the region of interest (ROI). Therefore, the approaches giving the good outcomes using the images of another modality (e.g., computed tomography) may not preserve them on the X-ray images.

Prior to the advent of DL, feature extraction was often manual and required knowledge from domain experts. In contrast, DL relies on neural networks to automatically learn effective feature representations via a nonlinear transformation of primitive data features, such as word vectors and picture pixels [15]. These DL-based algorithms are not trained to detect and classify anomalous lesions by inputting information about their shape, size, structure, and other characteristics. They are self-trained and consider various lesion parameters using a large image database for the training [16].

One of the most sophisticated DL networks is the convolutional neural network (CNN). CNN is a type of multilayer perceptron using the convolution operations. The works [17,18,19] show that CNN-based CAD systems for breast lesion detection and recognition achieve higher performance on the selected sets than conventional machine learning methods. YOLOv4 represents the CNN-based one—stage detector, which shows good accuracy in detecting lesions on a mammogram [20,21,22,23] with the accuracy in the range 80–95% in different databases (CBIS-DDSM, INbreast, etc.).

Previously, we developed CAD system based on the nested contours algorithm (NCA) especially designed for the X-ray image analysis that provided clinically significant results. Presently, the achievements in the field of deep learning and convolutional neural network technology provide the excellent opportunities to look at the problem of breast lesion detection and classification in a new way. Therefore, we tried to train the YOLOv4-based CNN and compare the results directly to the ones obtained with the help of the NCA.

## 2. Materials and Methods

### 2.1. Methods

#### 2.1.1. NCA

A description of NCA was already made in [24]. NCA is specifically developed to analyze 2D projections (mammography mass) of the 3D objects (breast lesion), which is its main feature. NCA requires no image pre-processing and uses the source image as an input. At first, level sets are built for the entire brightness range of the mammography image with a constant step of gradation *k*. From this set of level lines (contours), the ones nested to each other are sequentially identified. If the contour is different from the nested one obtained in the previous step gradation *k*, it is concluded that the contour under consideration does not represent a mass and should be discarded. The NCA demonstrated high sensitivity not only for the typical and clearly visible lesions, but also in the cases of atypical and poorly invisible changes (for example, asymmetric areas and invisible breast cancer obscured by the dense parenchyma background).

#### 2.1.2. YOLOv4

**Figure 1:**
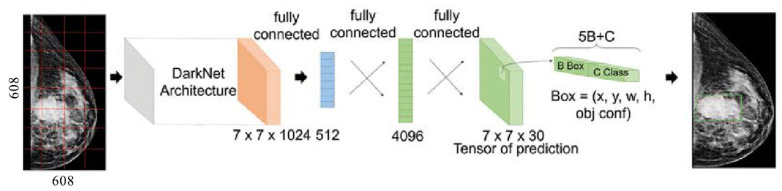
The YOLOv4 architecture with DarkNet framework.

The Yolo method is a one-stage detector that does not use a separate algorithm to generate regions, but instead predicts the coordinates of a certain number of bounding boxes with different characteristics, such as classification results and confidence levels, and then adjusts the location of the boxes. The YOLO architecture is based on the fully convolutional neural network (FCNN) construction. This approach splits each full image to the nets *N* × *N*, and for each net returns *B* limiting frames with the assessment of both significance and probability of the class *C* [25]. Figure 1 shows the implemented YOLOv4 architecture, where the DarkNet architecture is located at the input level. The DarkNet is an open source neural network framework written in C and CUDA.

To train the YOLOv4 model, we used 106 images from the INBreast dataset [26] containing two different mammographic types of BC: mass and microcalcification cluster. The INBreast dataset represents the wide variability of cases and is made publicly available with precise annotations.

We also added to the INBreast dataset 29 proven BC images where all the pathologic areas were segmented by the certified radiologist. Thus, in total we used images of 135 BC cases for the attempt of YOLOv4 training.

Most of the collected datasets have a small number of samples for medical applications and often suffer from an imbalanced distribution. To overcome this problem, two solutions were employed in many studies: data augmentation and transfer learning.

Because of the small size of the training set, we augmented the data. The augmented data were used for the training set only. Every image was multiplied eight times with random rotation, mirroring, and shift. Thus, we obtained 1080 images of BC. Additionally, for the YOLOv4, we used another approach for data augmentation, the Mosaic method [25]. The Mosaic method improves the generalization of object detection tasks. The Mosaic method represents a data augmentation method that mixes four training images. The method can better enrich the background of the target and prevent the degradation of the network generalization ability due to the similar background of the training set. In addition, the YOLOv4 has a strong generalization ability because it can learn highly generalized features to be transferred to other fields.

To improve the YOLOv4 results, we used the image pre-processing method [19]. This approach includes three steps:

1. Truncation normalization—according to the intensity histogram of the ROI image, a pair of the effective maximum intensity and minimum intensity is being selected, and then they are used to cut off the intensity of the image and finally to perform the normalize operating. This ensures that the breast region has a sufficient range of intensity distribution.
2. Image enhancement—Contrast limited adaptive histogram equalization (CLAHE algorithm) [27];
3. Image synthesizing—a 3-channel image is synthesized and composed of the truncated and normalized image, the contrast enhanced image with clip limit 1, and the contrast enhanced image with clip limit 2.

**Figure 2:**
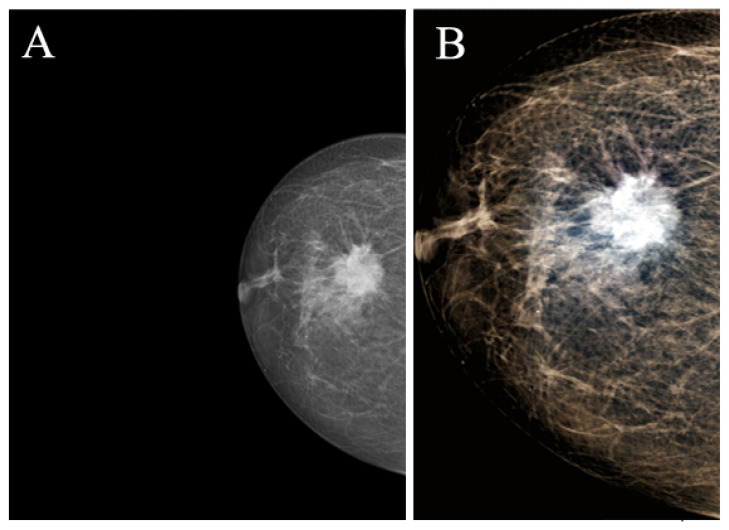
The result of the source image pre-processing. (**A**): source image; (**B**): after pre-processing.

Figure 2 shows the mammography image pre-processing result.

The dataset was split into two subsets to train YOLOv4: the training subset included 90% of the images and the validation subset included 10% of the images. We used the graphic processor NVIDIA Tesla K80 (16 Gb memory) to train the YOLOv4. The size of the input image was 608 × 608.

Since the dataset is currently limited, the model YOLOv4 was trained with the transfer learning method, where initial pre-trained weights learned on the Microsoft Common Objects in the Context (MS COCO) dataset were used [28]. The MS COCO dataset is a large-scale object detection, segmentation, key-point detection, and captioning dataset, which consists of 328 K images [29].

It is shown that the concept of transfer learning is effective in training a deep net for mammography images in [22,30]. In transfer learning, usually the last few layers of the network are replaced by new layers and initialized with random weights, the unchanged layers can be either frozen, i.e., made untrainable, or kept trainable. However, we perform training from the first layer and do not freeze some layers.

In the tasks of classification with localization and object detection, the ratio of the areas of the bounding boxes (Intersection over Union) is most often used as a metric to determine the reliability of the bounding box location:

$$IoU=\frac{S(A\cap B)}{S(A\cup B)},$$

where *A* and *B* are the predicted bounding box and the ground truth bounding box, respectively. *IoU* equals zero for non-overlapping bounding boxes and one for perfect overlap. In our case, average *IoU* was 80.05%.

In addition, in object detection tasks the mean Average Precision (*mAP*) is used as a metric, i.e., as a value of average precision over all categories:

$$AP=\overset{1}{\underset{0}{\int }}p(r)dr$$

where *p* is Precision, *r* is Recall based on the assumption that the bounding box is defined correctly, if *IoU* ≥ 0.5. Since Precision and Recall are between 0 and 1, then *AP*, hence, *mAP* is also between 0 and 1. In practice, *AP* is often calculated by points at which Recall values are evenly distributed in the interval [0;1]:

$$AP_{c}=\frac{1}{11}\cdot (AP_{c}(0)+AP_{c}(0.1)+\ldots +AP_{c}(1)),\;AP=\bar{A}{\bar{P}}_{c}$$

**Figure 3:**
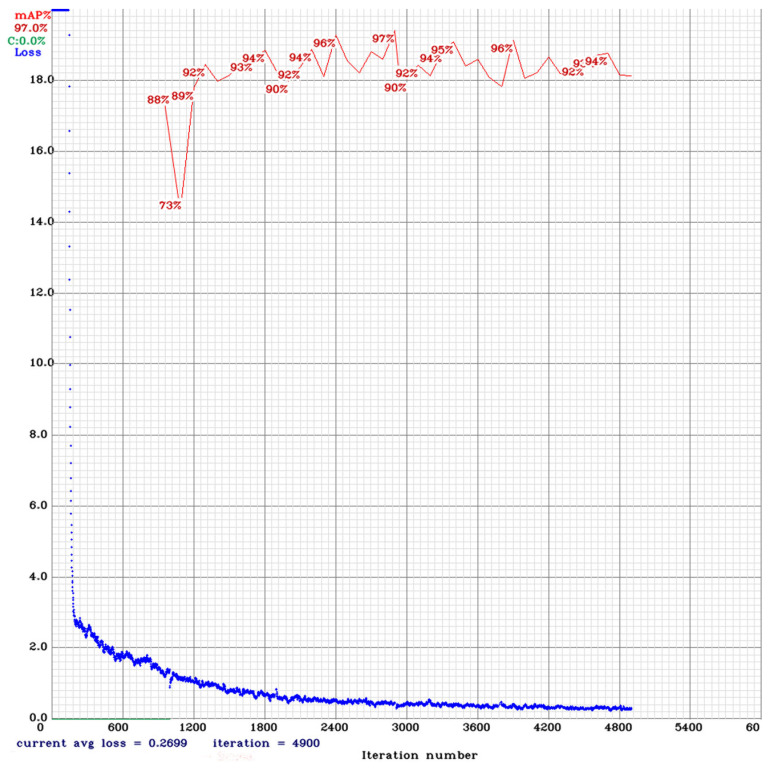
The results of the YOLOv4 training. The red line—mean Average Precision (*mAP*). The blue line–error graph (Loss).

Thus, when training in more than 4000 iterations, we obtained *mAP*_0.5_ = 96.23% (with the *IoU* threshold 50%). With the confidence threshold 0.25: Precision = 0.96, Recall = 0.91, *F*_1_-score = 0.93. Figure 3 shows the results of the YOLOv4 training.

Overfitting occurs when the accuracy on the training set keeps increasing while the accuracy of the validation set is decreasing between the epochs. The loss value of the models gradually decreased and the training of the model was stopped before the overfitting occurred.

### 2.2. Materials

After the training the YOLOv4 model, both the YOLO model itself and the NCA were tested on the test set that we did not use the YOLO for the training procedure.

The test set included 100 mammography images with the proven BC and 100 mammography images with the proven absence of BC. The distribution of mammographic BC types is shown in Table 1, and the density distribution is given in Table 2.

The rate of false-positive markings assessed in the subset of the images contained the proven BC; the rate of false-negative markings assessed in the subset of images contained the proven absence of BC.

### 2.3. Statistical Methods

To assess the results, the following three statistical categories were selected: True-Positive (*TP*; the model detected a lesion where it actually exists); False-Positive (*FP*; the model detected a lesion where it actually does not exist); False-Negative (*FN*; the model did not detect the lesion, where it actually exists). In addition, the accuracy, sensitivity, precision, recall, *F*_1_-score are often used to estimate the quality of mammogram lesion detection models [31,32,33,34,35].

We estimated the model performance by calculating Precision, Recall, and *F*_1_-score through these three categories of statistics. Precision shows the proportion of objects related to the lesions among the objects detected by the detector. Recall shows the proportion of the detected objects related to the lesions in the total number of lesion objects, i.e., how well our detector finds objects related to lesions. *F*_1_-score is the harmonic mean between Precision and Recall. We used Recall as the primary standard for identifying the version between the YOLO model and the NCA. Below are the mathematical definitions for Precision, Recall, and *F*_1_-score:

$$\mathrm{Precision}\;=\frac{TP}{TP+FP},\;\mathrm{Recall}=\frac{TP}{TP+FN},\;F_{1}-score=\frac{2\cdot \mathrm{Precision}\cdot \mathrm{Recall}}{\mathrm{Precision}+\mathrm{Recall}}.$$

Since detection of the BC regions is more important than several false-positive results, a weighted *F*_1_-score, i.e., *F*_β_-score, was also used. The *F*_β_-score measures the efficiency of the detector considering that Recall is β times more important than Precision:

$$F_{\beta }-score\;=\;(1+\beta^{2})\frac{\mathrm{Precision}\cdot \mathrm{Recall}}{\beta \cdot \mathrm{Precision}+\mathrm{Recall}}.$$

## 3. Results

**Figure 4:**
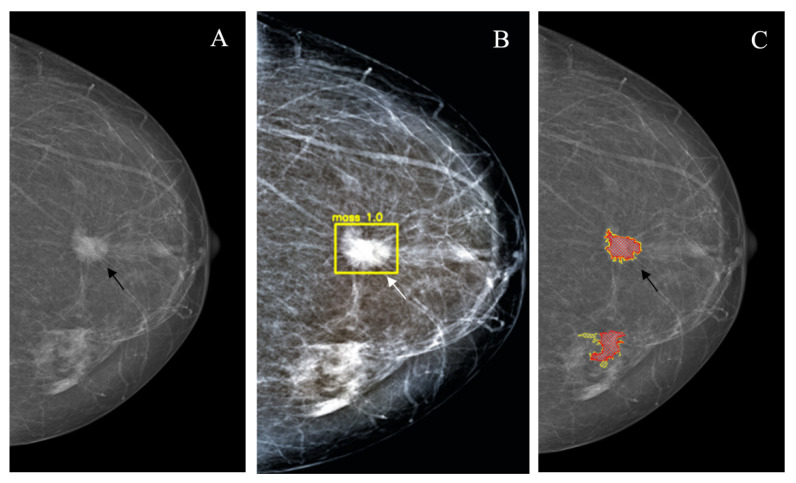
Star-like lesion (arrow). (**A**): Source image; (**B**): YOLOv4 outcome; (**C**): NCA outcome.

*Star-like lesion* (*n* = 16) represented the typical mammographic appearance of BC corresponding to the dense center with a long spicula. In most cases, these lesions corresponded to BIRADS 4–5 categories. The detection rate for the YOLOv4 and the NCA did not differ significantly; however, the rate of false-positive markings was significantly higher for NCA (9/16 vs. 0/16; *p* < 0.001) (Figure 4, Table 3).

**Figure 5:**
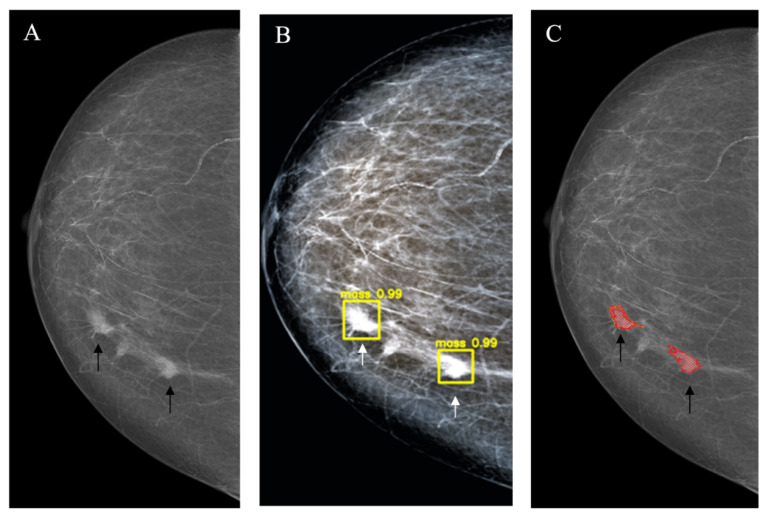
Mass with unclear border (arrows). (**A**): Source image; (**B**): YOLOv4 outcome; (**C**): NCA outcome. lesion (arrow).

*Mass with unclear border* had a less typical BC mammographic appearance, because the length of the spicula was lower compared to the star-like lesions. Therefore, they were usually described as BIRADS 3–4 lesions, and in some cases, it was not easy to distinguish them from benign masses. Nevertheless, in this situation, the sensitivities of the YOLOv4 and the NCA were also similar, but again the rate of false-positive markings was numerically higher for NCA (14/30 vs. 7/30; *p* = 0.059) (Figure 5).

**Figure 6:**
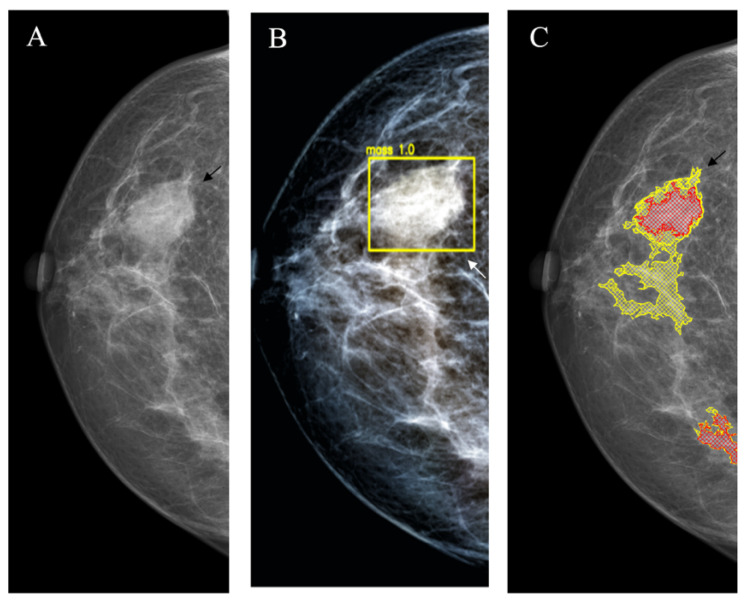
Round- or oval-shaped mass with clear border (arrow). (**A**): Source image; (**B**): YOLOv4 outcome; (**C**): NCA outcome.

*Round- or oval-shaped mass with clear border* in the majority of cases corresponds to the benign lesion; however, some malignancies (especially, mucinous carcinoma, lymphoma) may have similar characteristics. These lesions were rated as BIRADS 3. In this clinical situation, both approaches provided similar results with no difference in the rate of true and false-positive markings (Figure 6).

**Figure 7:**
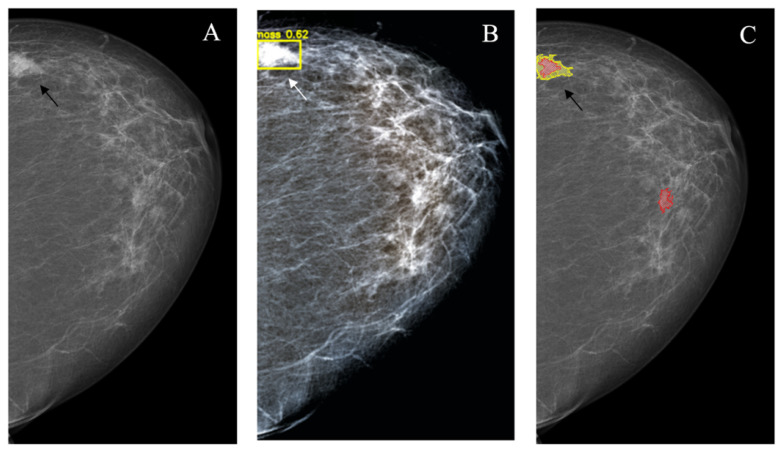
Partly visualized mass (arrow). (**A**): Source image; (**B**): YOLOv4 outcome; (**C**): NCA outcome.

*Partly visualized mass* was selected to belong to a separate class because its characteristics may become non-typical due to the fact that the certain part of the lesion is outside the field of view. In this case, both approaches provided similar outcomes; however, the number of cases was too small for meaningful interpretation (Figure 7).

**Figure 8:**
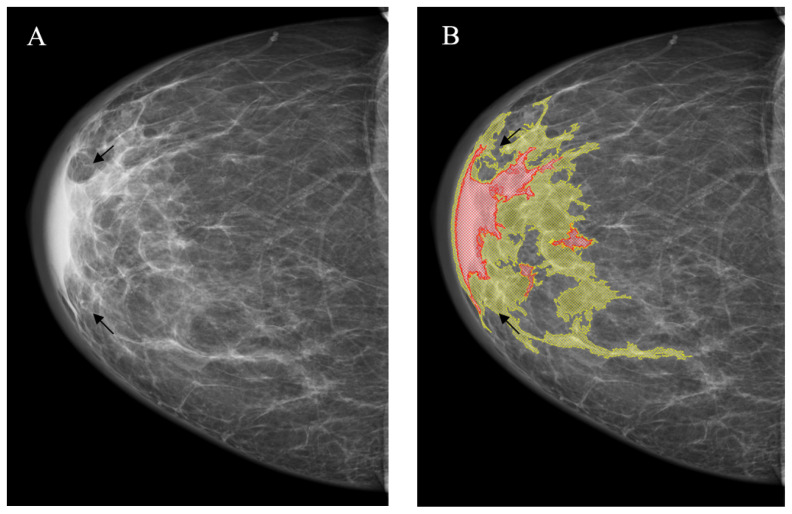
Asymmetric density (arrows). (**A**): Source image; (**B**): NCA outcome. The YOLOv4 did not mark the lesion.

*Asymmetric density* in the majority of cases also corresponds to the benign changes. At the same time, some BCs (approximately 2%), especially those surrounded by the dense parenchyma, may have a variable asymmetric mammographic picture. The YOLOv4 was able to mark 6 of 28 such lesions, which was lower compared to the NCA (27 of 28; *p* < 0.001). These results may be explained by the fact that the majority of asymmetric areas actually contain true focal lesions that are poorly visible on the hyperdense background and require the NCA approach for detection, because the textural and other characteristics assessed by the YOLOv4 are in distinguishable from those of similar benign areas. At the same time, the rate of false-positive markings was also higher for the NCA (18/28 vs. 0/28; *p* < 0.001) (Figure 8).

**Figure 9:**
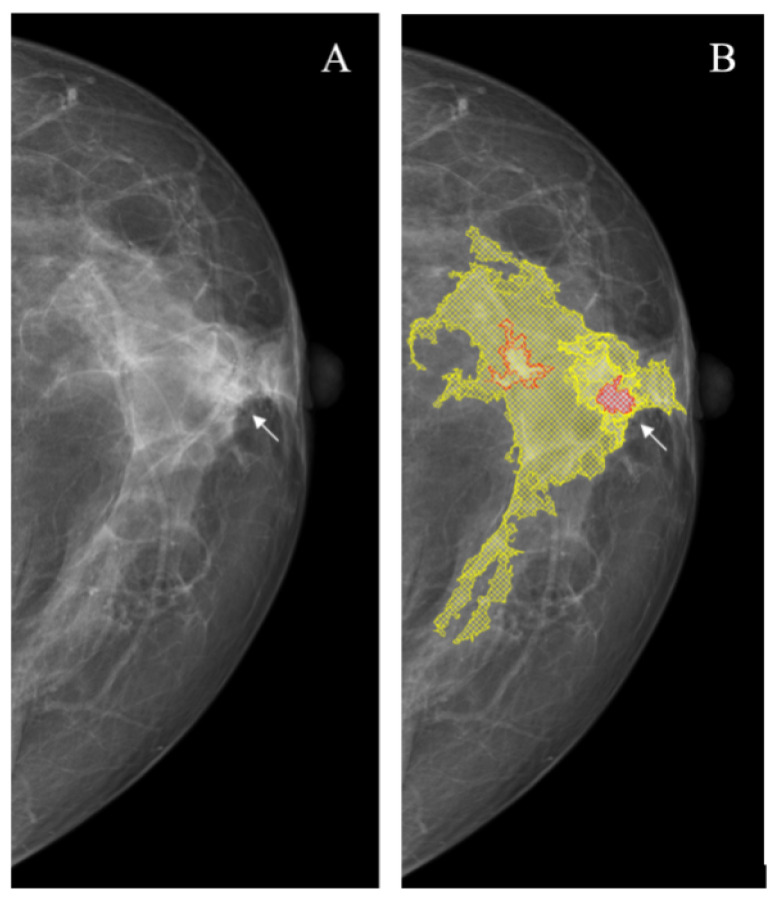
Changes poorly visible or invisible on the dense parenchyma background (arrow). (**A**): Source image; (**B**): NCA outcome. The YOLOv4 did not mark the lesion.

*Changes invisible on the dense parenchymal background* are the most clinically important problem of mammography because they are usually missed during the screening that significantly worsens the outcomes. This problem becomes even more important, because the capabilities of the clinical breast examination in this situation are also limited, and to improve the detection rate, other screening approaches are needed (e.g., ultrasound or tomosynthesis). In this situation, just like in the previous one, the NCA provided better sensitivity (16/16 vs. 5/16; *p* < 0.001) at the expense of the significantly higher rate of false-positive markings (16/16 vs. 0/16; *p* < 0.001) (Figure 9, Table 3).

To summarize, in our set the total sensitivity of the YOLOv4 was 60%, whereas for the NCA the total sensitivity was 93%.

**Figure 10:**
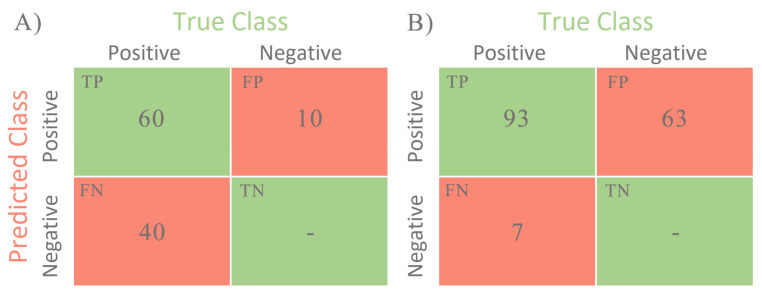
Confusion matrixes for (**A**): YOLOv4-based method and (**B**): NCA-based method. TP—the model detected a lesion where it actually exists; FP—the model detected a lesion where it actually does not exist; FN—the model did not detect the lesion, where it actually exists.

Figure 10 shows the obtained values of *TP*, *FP,* and *FN* for two compared approaches and their confusion matrixes.

Table 4 shows the values of Precision, Recall, *F*_1_-score for test set.

Table 5 shows the *F*_β_-scores for different significance values of β.

The presented results show that the use of the NCA method for lesion detection on mammograms is more significant, since it misses clinically important lesions to a lesser extent. A further use of additional soft filters can reduce the number of false-positive results.

In addition, the radiologists, to whom all the CAD markings were demonstrated, changed their decision about the case (additional examinations were performed) based on the CAD output in 6/100 cases for the NCA. The YOLOv4 outputs did not influence the radiologist’s decision.

## 4. Discussion

The YOLOv4 was already used for the mass-scale detection of the pre-processed images. It represents a one-step detector that is especially effective compared to the two-step detector in the cases where the context is necessary. It was demonstrated that in cases where the background does not depend on the foreground, the two-step detectors are useful, because the first step extracts the ROI. However, it was noted that for BC detection that is dependent on the breast parenchyma, the one-step detectors may be more effective [19].

Unlike the two-stage methods, in which in the first stage the regions of interest are determined by a selective search or a use of a special layer of a neural network, and in the second stage the selected regions are considered by a classifier to determine whether they belong to the original classes and a regressor that refines the location of the bounding boxes, the Yolo detector analyzes the entire image without splitting it into regions. This allows more accurate object recognition. Two-stage methods cannot consider global information, since the regional candidates are generated first and then feature extraction is being performed. Therefore, the global information cannot be taken into account when performing classification regression, and many false-positive results are possible. For example, Fast R-CNN (the best detection method [36]) marks background spots by mistake because it cannot analyze a wider context. Additionally, YOLO can generalize the representations of various objects that makes it more applicable to a variety of new environments. When trained on natural images and tested on artwork, the YOLO is vastly superior to the best detection methods, such as DPM and R-CNN. Since the YOLO is highly generalizable, it is less likely to break down when applied to new domains or unexpected inputs [37].

From the viewpoint of BC detection during the population-wide screening, the CAD sensitivity appears to be a more important indicator than the specificity due to the following observations described next. (1) mammography is usually the first step in BC detection where the majority of the suspicious changes are found; their discrimination into the requiring biopsy and follow-up is usually performed on the next step, for which the specificity of discrimination is more important. (2) mammography itself is not specific enough to provide sufficient lesion discrimination; therefore, it is hardly possible to significantly increase its specificity with the help of the CAD.

Moreover, CADs are most important for clinical practice in the cases when they detect poorly visible and invisible BCs (asymmetric areas, architectural distortions, etc.). On the other hand, the majority of the mass lesions are clearly visible on the low-density fatty background. This phenomenon is reflected by the data about the rate of radiologist’s decision change where the YOLOv4 had no such influence. For these mammographic BC types, the NCA clearly surpasses the YOLOv4 that makes it much more important despite the higher rate of false-positive markings, because they may be assessed by other modalities (e.g., by ultrasound).

What concerns the false-positive markings of the NCA is that the majority of them corresponds to the typical benign lesions (as for the YOLOv4); segmented areas of the dense parenchyma and Cooper ligaments projection crossings may be easily rejected by the visual analysis or the specific filtering.

One more point is in the CAD markings themselves. The NCA draws the approximate contour of the lesion, which may be preferential for the future visual analysis of the mark as well as for its comparison with the source image. On the other hand, the YOLOv4 shows the rectangle that contains the suspicious lesion, and in some cases, in which the contour of the lesion is not clear, it is more difficult to assess this area visually.

## 5. Conclusions

The NCA, based on the specific radiomic-based approach for mammographic images, was superior to the YOLOv4 in the cases of asymmetric density and invisible changes on the dense parenchyma background, which is clinically significant. On the other hand, YOLOv4 generates fewer false-positives. Therefore, it seems reasonable to combine both YOLOv4 and NCA to improve the quality of mammographic image classification in the future studies.

## Figures and Tables

**Table 1:** The distribution of the mammographic BC types in the test set.

Mammographic Type	*N*
Star-like lesion	16
Mass with unclear border	30
Round- or oval-shaped mass with clear border	8
Asymmetric density	28
Changes invisible on the dense parenchyma background	16
Partly visualized mass	2
Total	100

**Table 2:** The density distribution of all BC images included to the test set.

ACR Density Category	*N*
ACR * A	27
ACR B	33
ACR C	31
ACR D	9
Total	100

* Note: ACR = American College of Radiology.

**Table 3:** The rate of true-positive and false-positive outcomes for YOLOv4 and NCA-based CADs.

Lesion Type	True-Positive Markings		False-Positive Markings	
	YOLOv4	NCA	YOLOv4	NCA
Star-like lesion	15/16	16/16	0/16	9/16
Mass with unclear border	24/30	24/30	7/30	14/30
Round- or oval-shaped mass with clear border	8/8	8/8	3/8	4/8
Asymmetric density	6/28	27/28	0/28	18/28
Changes invisible on the dense parenchyma background	5/16	16/16	0/16	16/16
Partly visualized mass	2/2	2/2	0/2	2/2
Total	60/100	93/100	10/100	63/100

**Table 4:** Values of Precision, Recall, and *F*_1_-score.

Score	YOLOv4	NCA
Precision	0.85	0.59
Recall	0.60	0.93
*F*_1_-Score	0.70	0.72

**Table 5:** Values of *F*_β_ at different significance values of β.

β	YOLOv4	NCA
10	5.66	8.11
50	29.59	45.09
100	59.58	91.56
